# Probing three-dimensional sodiation–desodiation equilibrium in sodium-ion batteries by *in situ* hard X-ray nanotomography

**DOI:** 10.1038/ncomms8496

**Published:** 2015-06-26

**Authors:** Jiajun Wang, Christopher Eng, Yu-chen Karen Chen-Wiegart, Jun Wang

**Affiliations:** 1 National Synchrotron Light Source II, Brookhaven National Laboratory, Building 743 Ring Road, Upton, New York 11973, USA

## Abstract

Materials degradation—the main limiting factor for widespread application of alloy anodes in battery systems—was assumed to be worse in sodium alloys than in lithium analogues due to the larger sodium-ion radius. Efforts to relieve this problem are reliant on the understanding of electrochemical and structural degradation. Here we track three-dimensional structural and chemical evolution of tin anodes in sodium-ion batteries with *in situ* synchrotron hard X-ray nanotomography. We find an unusual (de)sodiation equilibrium during multi-electrochemical cycles. The superior structural reversibility during 10 electrochemical cycles and the significantly different morphological change features from comparable lithium-ion systems suggest untapped potential in sodium-ion batteries. These findings differ from the conventional thought that sodium ions always lead to more severe fractures in the electrode than lithium ions, which could have impact in advancing development of sodium-ion batteries.

## Abstract (web summary)

*In situ* 3D visualization of sodium-ion battery processes is challenging due to the highly active sodium metal and the sluggish kinetics. Here, the authors present a X-ray tomography technique, which enables tracking the sodiation–desodiation process of a Sn anode in battery operation.

## Graphical abstract

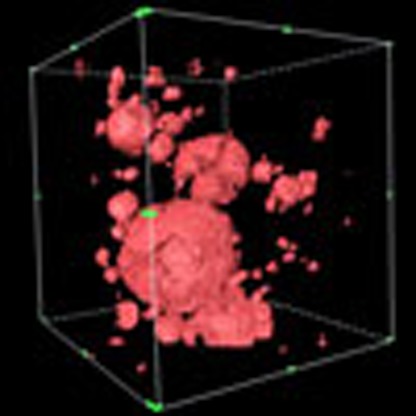

Though they share similar properties and principles, the nature of sodium (Na)-ion batteries (NIB) has not been explored as extensively as lithium (Li)-ion batteries (LIB). Similar to its Li-ion analogues, some metal anodes can electrochemically store/release Na ions with an alloying and dealloying mechanism, exhibiting high theoretical-specific capacity^1^,^2^,^3^,^4^,^5^,^6^,^7^. Nevertheless, the larger Na-ion radius may result in not only the larger volume change of battery materials, but also some possible discrepancies in behaviours between Na and Li analogue materials. Some previous strategies for Li-ion system may therefore not necessarily work for Na ions and *vice versa*. Unfortunately, compared with extensively studied Li alloy anodes, fundamental understanding of sodiation–desodiation process and the mechanical degradation are still unclear. *In situ* observation and tracking of the structural change and morphological evolution of battery materials provide information to directly explore the microstructural or chemical information^8^,^9^. Considering the three-dimensional (3D) electrode orientation and possible anisotropic electrochemical reactions, an *in situ* 3D visualization technique is highly valuable, but highly challenging for NIB due to the highly active Na metal and the sluggish kinetics. Compared with Li metal, the higher chemically active Na poses significant challenges for *in situ* electrochemical cells such as safety, sealing and durability. The slow Na-ion diffusion and electrochemical reaction also demand more time to complete electrochemical measurement and data collection/analysis (Supplementary Note 1).

X-ray tomography has recently attracted much attention as an emerging nanotechnology with escalating impact on energy materials research^10^,^11^,^12^,^13^,^14^,^15^. In spite of considerable advances, one main difficulty in 3D tomography reconstructions is the need for manual alignment of two-dimensional (2D) projections, which considerably affects 3D resolution, particularly for local regions of interest. At Brookhaven National Laboratory (BNL), a newly developed full-field transmission X-ray microscope (TXM) operated at X8C beamline, National Synchrotron Light Source (NSLS), has overcome the difficulty to provide automated and local tomography unique capabilities^16^. Sub-30 nm resolution in 2D and sub-50 nm resolution in 3D with markerless automated tomography have been achieved. In this work, by using this advanced full-field X-ray tomography technique^16^,^17^,^18^,^19^, after overcoming the technical/*in situ* experimental difficulties in NIB system, we successfully tracked the sodiation–desodiation process of Sn anodes during 10 electrochemical cycles. With detailed 3D quantitative analysis and correlating it to discharge/charge performance, we reveal a surprising structural/chemical equilibrium and electrochemical reversibility in Sn anodes for NIB systems.

## Results

### 3D microstructural evolution

**Figure 1:**
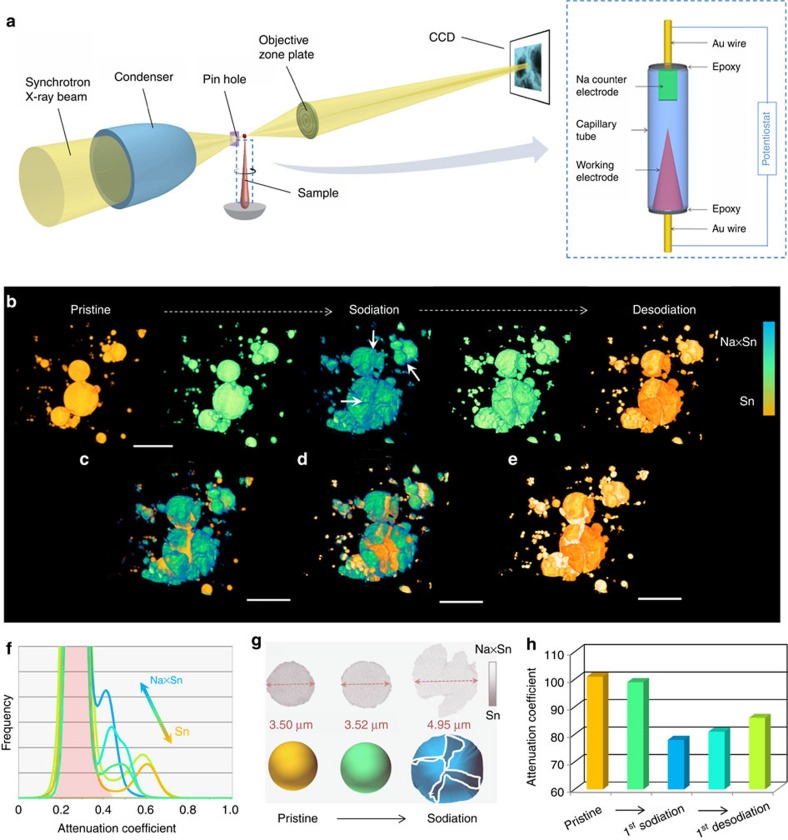
Visualization of three-dimensional (3D) microstructural evolution in NIB. (**a**) Experimental setup and electrochemical cells. (**b**) 3D morphological change of Sn particles during the first sodiation–desodiation cycle. The colours in **b** represent the attenuation coefficient variation within the reconstructed images. Overlapped 3D views of Sn particles at different electrochemical stages: (**c**) pristine/sodiated, (**d**) sodiated/desodiated and (**e**) pristine/desodiated. The contrast of the overlapping colors (**c**–**e**) are adjusted for better visualization. (**f**) Normalized X-ray attenuation coefficient histogram during the first electrochemical cycle. (**g**) Schematic illustration showing the two-step sodiation process, which is supported by the cross-section images of selected particle shown above (grey), in which the initial sodiation process leads to negligible volume change. (**h**)The histogram information of attenuation coefficient change, which is correlated the above 3D morphological change to chemical information change. Scale bar, 10 μm.

The *in situ* experimental setup and 3D structural evolution of Sn particles during the first electrochemical cycle are represented in Fig. 1a–e (Supplementary Figs 1 and 2, Supplementary Movies 1 and 2). At the beginning of the sodiation process, Sn particles undergo negligible volume expansion and the overall structures remain unchanged. With further sodiation, severe morphological expansion occurs and many cracks were observed (indicated by white arrows; Supplementary Figs 3 and 4). During the following desodiation process, the extraction of Na ions leads to the volume shrinkage, but surprisingly, no obvious pulverization or structural failure occurs. This modest structural damage during Na-ion extraction is remarkably different from the analogous LIB in which the Li-ion extraction rather than the insertion process evokes severe structural degradation and the formation of many pores^17^.

**Figure 2:**
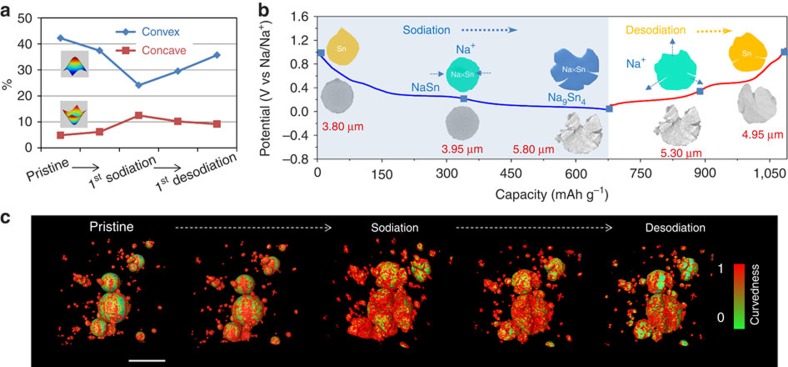
3D view and quantitative analysis of surface curvature evolution in NIB. (**a**) Evolution of the two principal curvatures, convex and concave, during the first electrochemical cycle. (**b**) Schematic illustration (colour) showing surface curvature change, and the corresponding real cross-section images (grey) of selected particle shown below. The discharge/charge profile is presented as the background. (**c**) 3D view of surface curvedness evolution during the first electrochemical cycle. The curvedness is calculated on the two principal curvatures, convex and concave feature. See Supplementary Information for details. Scale bar, 10 μm.

The chemical information change can be quantified with the linear attenuation coefficient^20^,^21^,^22^,^23^. For example, the chemical compositions of Li_*x*_Sn were identified by the attenuation coefficient change^21^. The X-ray attenuation of Si also decreases with lithiation, referring to the Si→Li_*x*_Si^22^. In this work, the decrease of the attenuation coefficient indicates the sodiation process from Sn to Na_*x*_Sn in NIB. The left high peak (pink background) in Fig. 1f corresponds to the background information (electrolytes, conductive carbon, binder and others), and the right peaks correspond to active materials (Sn or Na_*x*_Sn). The voltage curve shows three voltage plateaus (Supplementary Fig. 5), which refer to the Na–Sn alloying two-phase reactions of Sn–NaSn_5_ (∼0.5 V), NaSn_5_–NaSn (∼0.25 V) and NaSn–Na_9_Sn_4_ (∼0.15 V)^23^,^24^. The tomography data was recorded at the end of the second voltage plateaus, corresponding to a phase of Na_*x*_Sn (*x*>1.0). The attenuation coefficient change during the initial sodiation process also indicates the phase transformation. This chemical phase change was also confirmed by the discharge curve shown in Fig. 2. Intriguingly, no obvious morphological change was observed at this step, which indicates that the initial sodiation process, despite contributing to the specific capacity, shows negligible volume change. This feature may be attributed to some amorphous phase formation^25^, which is similar to the multi-step lithiation process in Si, Ge and Sn in LIB^26^,^27^,^28^. The carbon paper with matrix network structure may also buffer the volume expansion. During the further sodiation process, the voltage plateaus (0.15 V) and the big negative shift in attenuation coefficient suggest the formation of Na_9_Sn_4_ or even higher-sodiation Na_*x*_Sn (*x*≥2.25) phase, which is also revealed by the drastic morphology change shown in Fig. 1a. This sodiation process was also visualized by an *in operando* 2D TXM study. The setup was shown in Supplementary Fig. 6 and the morphological evolution was shown in Supplementary Fig. 7 (also see Supplementary Video 3). Despite the discharge plateau and the responding capacity referring to the chemical phase change, the Sn particles show no obvious diameter increase and morphology expansion at the beginning of sodiation. After that, with further sodiation, large morphology expansion and diameter increase happen. Therefore, based on the above studies, the sodiation process is initiated by the formation of a Na_*x*_Sn (*x*≈1.3, see Supplementary Note 2) phase with low volume change, and followed by further Na-ion insertion that increasing sodiation-induced stress leads to volume expansion/fracture (Fig. 1g).

During the desodiation process, a positive shift at the attenuation coefficient indicates the phase transformation from Na_*x*_Sn (*x*≥2.25) to Sn. Nevertheless, the attenuation coefficient differs from the initial value of Sn (Fig. 1h), suggesting that the desodiation process is not completely electrochemically reversible, which is consistent with the irreversible morphological change and the charge/discharge capacity. This is because some Na ions may be trapped inside the Na_*x*_Sn microstructure, and cannot be completely extracted out during the first desodiation process. As evidenced by the cyclic voltammogram (CV) measurement in the Supplementary Fig. 8, the several peaks at the first cathodic scan (except the peak at 0.9 V that corresponds to solid electrolyte interphase layer formation due to electrolyte decomposition) and the corresponding peaks at the anodic scan can be assigned to the phase transformation of Sn→Na_*x*_Sn and Na_*x*_Sn→Sn, respectively^25^. Obviously, from these CV curves, the overall intensity and the integrated capacity of the anodic peaks are lower than that of the cathodic peaks, indicating the partial irreversibility at the first sodiation–desodiation process.

In addition to the partially irreversible behavior, a remarkably different feature from the analogous LIB is that, in spite of significant volume shrinkage, negligible structural pulverization/damage was observed after the first Na-ion extraction; whereas in a LIB, mechanical degradation with drastic morphology change (pores, pulverization) of anode materials occurs mainly during the first delithiation process, not the first lithiation. This relatively mild structural change at the initial sodiation–desodiation cycle may favour the microstructural stability in the following electrochemical cycles.

### 3D quantitative analysis

It is well-known that materials degradation is directly correlated to the change in their geometric characteristics during electrochemical cycling^30^,^31^. The distributions of the two representative principal curvatures, which can be used to identify convex and concave surface, are evaluated on a statistical analysis of all particles (Fig. 2a, Supplementary Fig. 9, Supplementary Table 1, Supplementary Note 3). At the beginning of Na insertion, convex surface slightly decreases and concave surface increases (Fig. 2b), but the overall volume remains stable. The further Na insertion result in large lattice mismatch between Sn and Na_*x*_Sn. The enormous strain at the alloying front is subsequently released by the formation of many cracks and fractures at the end of sodiation process, leading to concave feature increase. At the desodiation process, the Na-ion extraction induces volume shrinkage and convex surface increase, but the overall microstructure maintains the integrity with negligible pulverization. The 3D surface view of curvature (Fig. 2c) computes the surface scalar field, which is based on the two principal curvatures. The increasing surface curvedness during sodiation and decreasing surface curvedness during the desodiation process directly reveal the microstructural change, as the large amount of high-curvature small objects are created by the materials' pulverization/damage after Na insertion, whereas the volume shrinkage on Na-ion extraction merges these small objects to form low-curvature large particles. So, this 3D surface view, along with the 2D experiment (Supplementary Figs 10 and 11), clearly confirms that the microstructural damage mainly occured at the sodiation process.

**Figure 3:**
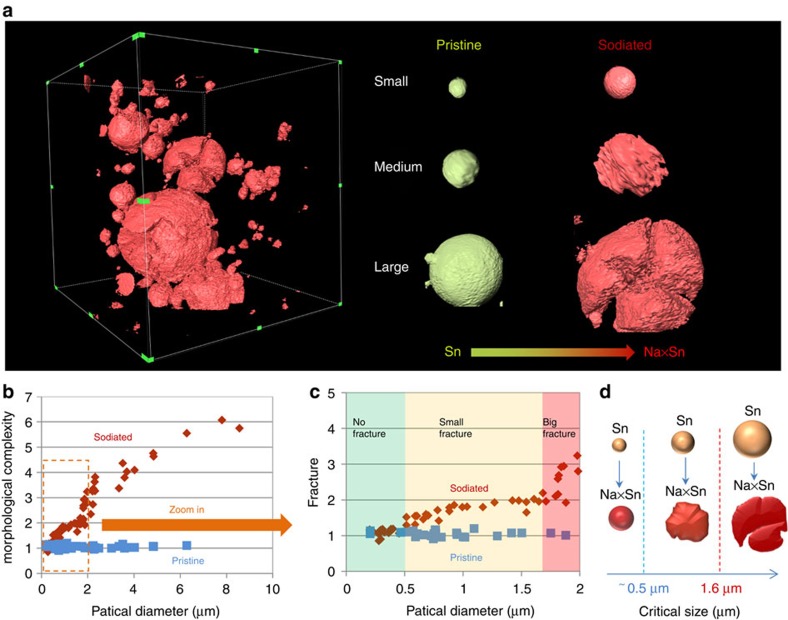
Statistical analysis of morphological complexity degree. (**a**) 3D morphologies of the sodiated electrode and selected three particles with different size and fracture. (**b**) Size-dependent morphological complexity for the pristine and sodiated samples. (**c**) An enlarged plot showing three different fracture zone. (**d**) A schematic illustration gives the two critical sizes for Sn fracture in NIB.

To quantify the structural failure in NIB, statistical analysis of morphological complexity degree were performed on these individual particles. The 3D morphologies of the entire electrode and several representative particles (small, medium and large sized) were shown in Fig. 3a. Here the complexity degree (a unit-less parameter, *η*) is measured by the ratio of the radius *R*_s_ (the radius calculated from the actual surface area) to the radius *R*_v_ (the radius from the volume *V*; Supplementary Note 4). If an ideally ball-shaped particle (most of pristine Sn particles are ball-shaped, as shown in the mosaic image) is robust enough to survive the big volume expansion (no cracks, fracture or pulverization) after Na ions insertion/extraction, the *η-*value should be around 1. The higher the value is, the more complexity has occurred. This particle complexity can refer to the microstructural degradation in pores, cracks, fracture and pulverization. The similar parameter was also used to quantify the microstructural change for cathode battery materials that generally low-morphological change exist^32^. The statistical result in Fig. 3b shows an obvious size-dependent complexity for Sn particles, and the zoom-in plot (Fig. 3c) gives three different complexity zones, in which low complexity occurs above 0.5 μm zone and high complexity happens over 1.6 μm zone. Below 0.5 μm, Sn particles show negligible structural degradation and keep the microstructural integrity. Therefore, the two critical sizes (0.5 μm for low complexity and 1.6 μm for high complexity, Fig. 3d) provide new insights into the failure mechanism and a basis for further engineering battery materials in NIB.

### The (de)sodiation equilibrium during 10 electrochemical cycles

**Figure 4:**
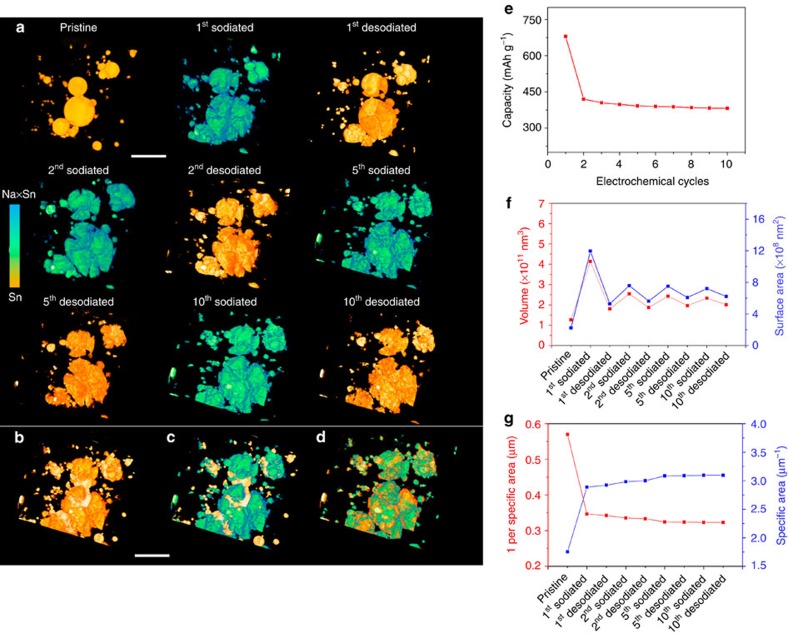
3D view of microstructural equilibrium in NIB. (**a**) microstructural reversibility during 10 sodiation–desodiation cycles; The overlapped images of (**b**) pristine/second desodiated, (**c**) pristine/tenth sodiated and (**d**) second sodiated/tenth desodiated. The contrast of the overlapping colors (**b**–**d**) are adjusted for better visualization. A microstructural equilibrium reaches since the second sodiation–desodiation process. (**e**) The electrochemical cycle performance; (**f**) volume and surface area change during the electrochemical cycles; (**g**) quantitative specific area change during the electrochemical cycles. Scale bar, 10 μm.

In spite of significant morphological change during the first cycle, Sn particles seem to show excellent microstructural stability during the following cycles (Fig. 4a–d). The high mechanical stability is well consistent with excellent electrochemical reversibility (Fig. 4e), which is further supported by the 3D quantitative analysis (Fig. 4f,g). The volume expansion is ∼326% after the first sodiation, which will lead to an increase of surface area by a factor of ∼2.19 if there is no morphological degradation. But the overall surface area undergoes five times increase, which suggests that significant microstructural degradation occurred resulting in the formation of many small objects during the first sodiation. The specific surface area (defined as the surface area per unit volume of materials), a typical structural parameter to characterize material's pulverization, also reveals the same trend. Interestingly, since the second cycle, both volume and surface area undergo reversible expansion and shrinkage, and the specific surface area keeps stable, indicating that the microstructure reaches equilibrium.

**Figure 5:**
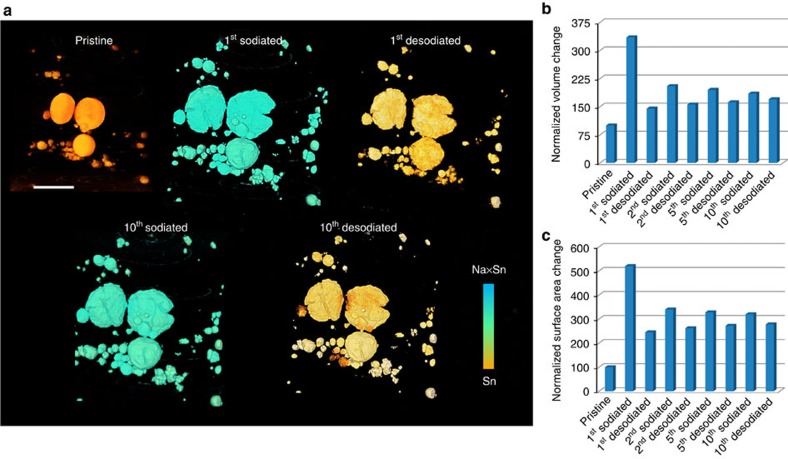
3D microstructural equilibrium of another Sn electrode in NIB. (**a**) 3D morphologies of the Sn electrode during 10 electrochemical cycles. (**b**) The statistical analysis of volume change during the 10 cycles. (**c**) The statistical analysis of surface area change during the ten electrochemical cycles. Scale bar, 10 μm.

We performed the same *in situ* 3D experiment on a new sample to further confirm the surprising (de)sodiation equilibrium and microstructural reversibility. The glavanostatic discharge–charge profiles and cycler performance were shown in Supplementary Fig. 12, demonstrating the reversible sodiation–desodiation after the first cycle. Figure 5 shows the 3D morphological information during the 10 electrochemical cycles. The colours were shown based on the attenuation coefficient variation within the reconstruction images, indicating the phase change between Sn and Na_*x*_Sn. Similar to the results in Fig. 4, after the microstructural degradation during the first cycle, the particles reach a structural equilibrium and mechanical reversibility, as shown in the tenth sodiated and desodiated sample. The 3D quantitative analysis of volume and surface area also shows the same trend. More information can be found at the mosaic images shown in Supplementary Figs 13 and 14.

**Figure 6:**
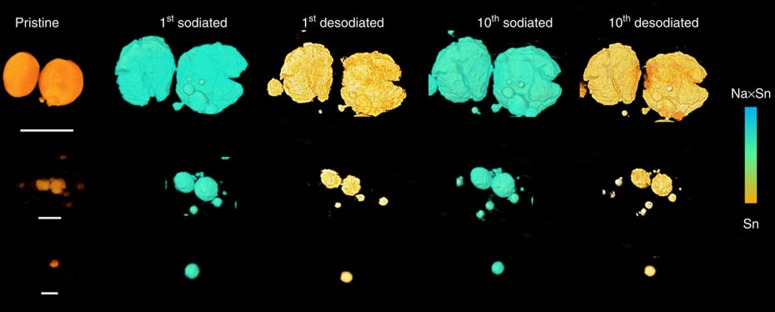
3D view of selected Sn particles with different size range. The Sn particles are listed as three size ranges (5–10 μm, ∼2 μm and <1 μm). Scale bars, 10, 2 and 1 μm.

To evaluate the size-dependent microstructural change, we selected three groups of representative particles with different size range (Fig. 6). For the smallest Sn particle (∼0.5 μm), no obvious microstructural degradation was observed but reversible expansion/shrinkage was observed during the 10 cycles. As for the medium size range (1–2 μm), mild microstructural degradation occurred, but for the larger particles (5–10 μm), significant structural change with large cracks was found (also see Supplementary Fig. 15 for more information). This result is consistent with the statistical analysis in Fig. 3b,c.

## Discussion

**Figure 7:**
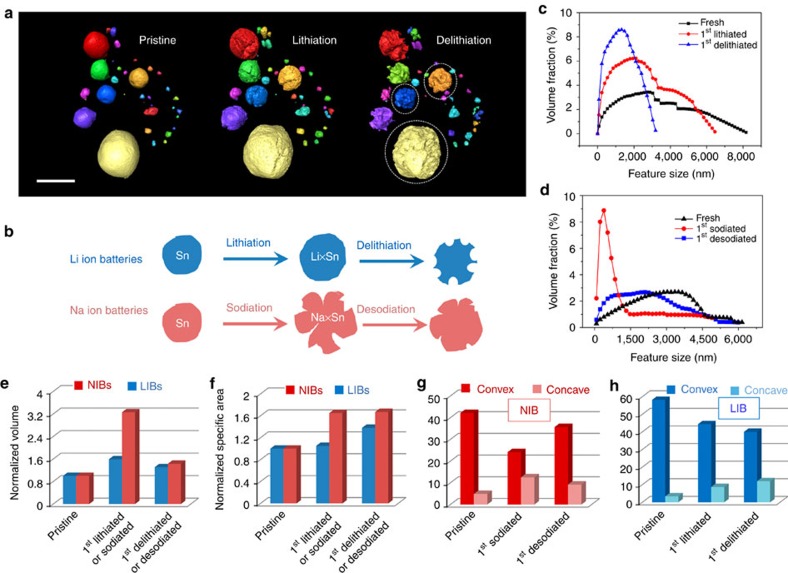
Comparison of Sn anodes in NIB and LIB systems. (**a**) 3D visualization of Sn particles at the first lithiation–delithiation process. Particles are coloured on particle size to assist in data analysis and view. (**b**) Schematic illustration showing the difference of Sn microstructural change in NIB and LIB. For NIB, the sodiation leads to obvious microstructural cracks, but the desodiation process only shrinks the volume with negligible pulverization. In contrast, the microstructural change in LIB predominantly occurs during the delithiation process, with the formation of many pores and pulverization. The feature size distribution of Sn particles in (**c**) LIBs and (**d**) NIBs confirms the sketch in **b**. (**e**,**f**) 3D quantitative comparison of (**e**) volume, (**f**) specific area, (**g**) curvature in NIBs and (**h**) curvature in LIBs. Scale bar, 10 μm.

Considering the larger ionic radius, the excellent structural stability of Sn in NIB is surprising, which inspired us to perform a comparative study. Compared with Sn in NIB, severe microstructural change accompanied with large amounts of pores mainly occurs at the delithiation process in LIB, not the lithiation step (Fig. 7a,b, Supplementary Movie 4). When Li ions are initially inserted into Sn anode, in spite of volume expansion, Li_*x*_Sn shows low microstructural damage, but Li-ions extraction induces enormous morphology pulverization, featuring many pores formation. In contrast, Na-ion insertion results in large volume expansion and fracture in NIB, whereas Na-ion extraction only induces the volume shrinkage with negligible pulverization and maintains microstructural integrity. This difference in microstructural change can be also revealed by the feature size distribution (Fig. 7c,d). The smallest feature size in LIB corresponds to the delithiated process, while it corresponds to the sodiated step for NIB. Despite Sn anodes showing larger volume expansion in NIBs (Fig. 7e), the specific area parameter suggests a microstructural equilibrium after the first sodiation (Fig. 7f). To understand the origin of microstructural equilibrium, the surface curvature evolution was illustrated in Fig. 7g,h. Since concave features play a major role in affecting the microstructural stability, the continuous increase at the both lithiation and delithiation process indicates that the microstructure cannot reach equilibrium after the first cycle for LIB, so further microstructure damage may continue during the following cycles. In contrast, the significant increase after the sodiation process and the following decrease after the desodiation step for the concave features indicate that the microstructures reach mechanical equilibrium at the end of the first sodiation. As a result, the superior electrochemical stability at the following cycles can be obtained in NIBs. It should be noted that this comparative study is to show the significantly different morphological features of Sn in NIB and LIB. No cycle performance is directly compared in this work. It should be mentioned that this *in situ* 3D study and the quantitative analysis are based on X-ray imaging technology to provide microstructural information in 3D and reveal the large-scale electrode behavior, which can represent the entire battery electrode. Some nanostructural change may also exist at higher resolution scale which is beyond the existing hard X-ray TXM imaging resolution limitation^33^,^34^, and is not discussed in this work. Technological advances in lensless coherent diffraction imaging method can achieve higher spatial resolution, and will be potentially applied in nanostructural materials research^35^.

In summary, by tracking 3D structural/chemical evolution of Sn during multi-electrochemical cycles, we uncover a superior (de)sodiation equilibrium in NIB systems. The 3D microstructural reversibility and curvature feature development explain the exciting structural equilibrium, which enable achieving the high-electrochemical cycle stability in NIB. We also suggest two important critical sizes (0.5 and 1.6 μm) for materials' fracture in NIB at this scale. Our findings provide valuable information about (de)sodiation kinetics and the failure mechanism of NIB during multi-electrochemical cycles. The unexpected chemical/structural equilibrium and different morphological change features of Sn anodes in NIB and LIB indicates that it is urgent to revisit some LIB-type electrode materials for NIB. The study of morphological evolution, surface curvature and electrochemical performance provides valuable information for better understanding the failure mechanisms in these battery systems, serving as a new strategy to address the problem of sodiation-induced stress in NIB electrodes. This *in situ* methodology with 3D imaging and accurate quantitative analysis is broadly applicable for other energy materials.

## Methods

### Assembly of micron-scaled Na-ion batteries

The battery is a closed system using Na metal as the counter electrode and 1 M NaClO_4_ (Sigma-Aldrich) in a mixture of ethylene carbonate/dimethyl carbonate (1:1 by volume, Sigma-Aldrich) as the electrolyte. Sn powder (Aldrich) mixed with acetylene black and polyvinylidene fluoride with a weight ratio of 50:35:15 in N-methyl-2-pyrrolidone (Sigma-Aldrich) solvent to produce the slurry. The resultant slurry, pasted on commercial carbon paper (Toray Carbon Paper TGP-H-030), was dried at 100 °C under vacuum for 24 h to form the working electrode. The Sn loading content is controlled to be ∼0.8 mg cm^−2^. The Sn electrode is cut into a trapezoid shape (∼ 40 μm short side, 800 μm long side, and 1 cm height) under optical microscopy (LEICA DM4000M) to meet the field of view (40 × 40 μm^2^) of the TXM. Based on Sn loading of ∼0.8 mg cm^−2^, the trapezoid shape working electrode (0.045 cm^2^) has 0.036 mg Sn loading. The mini Na-ion battery was fabricated in a quartz capillary (1 mm diameter) in an Ar-filled glove box, and sealed with epoxy. The two gold rods (0.5 mm diameter) connected with the two electrodes of the external potentiostat, completing the circuit.

Similar to conventional coin cells, the battery is a closed system where electrode materials were immersed completely in common liquid electrolytes to allow reactions in all dimensions. This unique design also allows performing a variety of electrochemical measurements such as cyclic voltagram and discharge/charge profile because large amount of active materials in this mini battery provides high-enough current to meet the potentiostat sensitivity. Another advantage of this closed battery system is its stability, which allows long electrochemical cycling (over weeks) under X-ray characterization.

### *In situ* nanotomography with TXM

The mini battery was then imaged using TXM at beamline X8C, NSLS (BNL). This newly developed TXM at BNL is capable of markerless, automated tomography. Because of the unique capability of the local tomography offered in the experiment, the 3D morphology of the volume of interest within the cell could be reconstructed even though the particles were enclosed in the *in situ* electrochemical cell. For each electrochemical sodiation–desodiation stage, a nanotomography data set was collected with 8 keV X-rays, using 361 projections over an angular range of 180° with a field of view of 40 × 40 μm^2^ (with a 2 k × 2 k CCD (charge-coupled device) camera binning 2 × 2 camera pixels into one output pixel). The pixel size was 38.9 nm. The resolution of this TXM has been quantified in our previous paper^16^, and a sub-50 nm 3D resolution was demonstrated in a LiCoO_2_ commercial electrode. This *in situ* 3D tomography work was carried out with 50–100 nm resolution at 3D. Before reconstruction, the raw data have been corrected with a run-out correction system build in the sample stages for an automatic tomography alignment available at beamline X8C, NSLS. The *in situ* electrochemical measurements were performed on a versatile multichannel potentiostat. Before electrochemical measurements, a high current pulse was initially applied to the cell until the voltage is below 0.8 V, so the electrolyte decomposition issue can be minimized. Charge–discharge characteristics were therefore galvanostatically carried out at a potential range between 0.005 and 1.0 V (vs Na/Na^+^) at room temperature. During the first charge/discharge cycle, in addition to the finial sodiated (0.005 V) and desodiated stage (1.0 V), the tomography data at the intermediate sodiated and desodiated (Fig. 1a) stages were also recorded. Then, during the 10 electrochemical cycles, a series of tomography data were recorded at the second, fifth and tenth sodiated/desodiated stages.

### *In operando* TXM experiment

The *in operando* 2D TXM experiment was performed on coin cells with Kapton windows^18^,^19^. Na metal was used as the counter electrode and 1 M NaClO_4_ in a mixture of ethylene carbonate/dimethyl carbonate (1:1 by volume) as the electrolyte. The above Sn electrode was used as the working electrode. The Na-ion batteries were assembled in a glove box, following the procedure of conventional Li-ion coin cells. To track the correlation of volume change to applied voltage, the sodiation–desodiation was achieved by two electrochemical measurements. One is CV between 0.005 and 2.5 V (or 1.0 V), and the other is charge–discharge characteristics at a potential range between 0.005 and 1.0 V (vs Na/Na^+^) at room temperature. A series of 2D images were recorded at 10 min interval with binning 2, exposure time 10 s setting.

## Additional information

**How to cite this article:** Wang, J. *et al.* Probing three-dimensional sodiation-desodiation equilibrium in sodium-ion batteries by *in situ* hard X-ray nanotomography. *Nat. Commun.* 6:7496 doi: 10.1038/ncomms8496 (2015).

## Supplementary Material

Supplementary Information Supplementary Figures 1-17, Supplementary Tables 1-2, Supplementary Notes 1-4 and Supplementary References

Supplementary Movie 1 An In situ TXM movie showing the 3D morphological evolution of Sn particles during the first sodiation-desodiation cycle.

Supplementary Movie 2 The overlapped 3D morphological evolution showing a detailed surface and microstructural change of Sn particles during the 1st sodiation-desodiation process.

Supplementary Movie 3 The in operando 2D morphology evolution of Sn particles during the 1st sodaition.

Supplementary Movie 4 3D morphological evolution of Sn particles during the first electrochemical cycle in a lithium-ion battery.
