# Supplementary material for: Probing three-dimensional sodiation–desodiation equilibrium in sodium-ion batteries by in situ hard X-ray nanotomography
Source: Nat Commun. 2015 Jun 26;6:7496. doi: 10.1038/ncomms8496 (PMC4491187; doi:10.1038/ncomms8496)
Supplement: Supplementary Information — Supplementary Figures 1-17, Supplementary Tables 1-2, Supplementary Notes 1-4 and Supplementary References [file ncomms8496-s1.pdf]

Supplementary Information

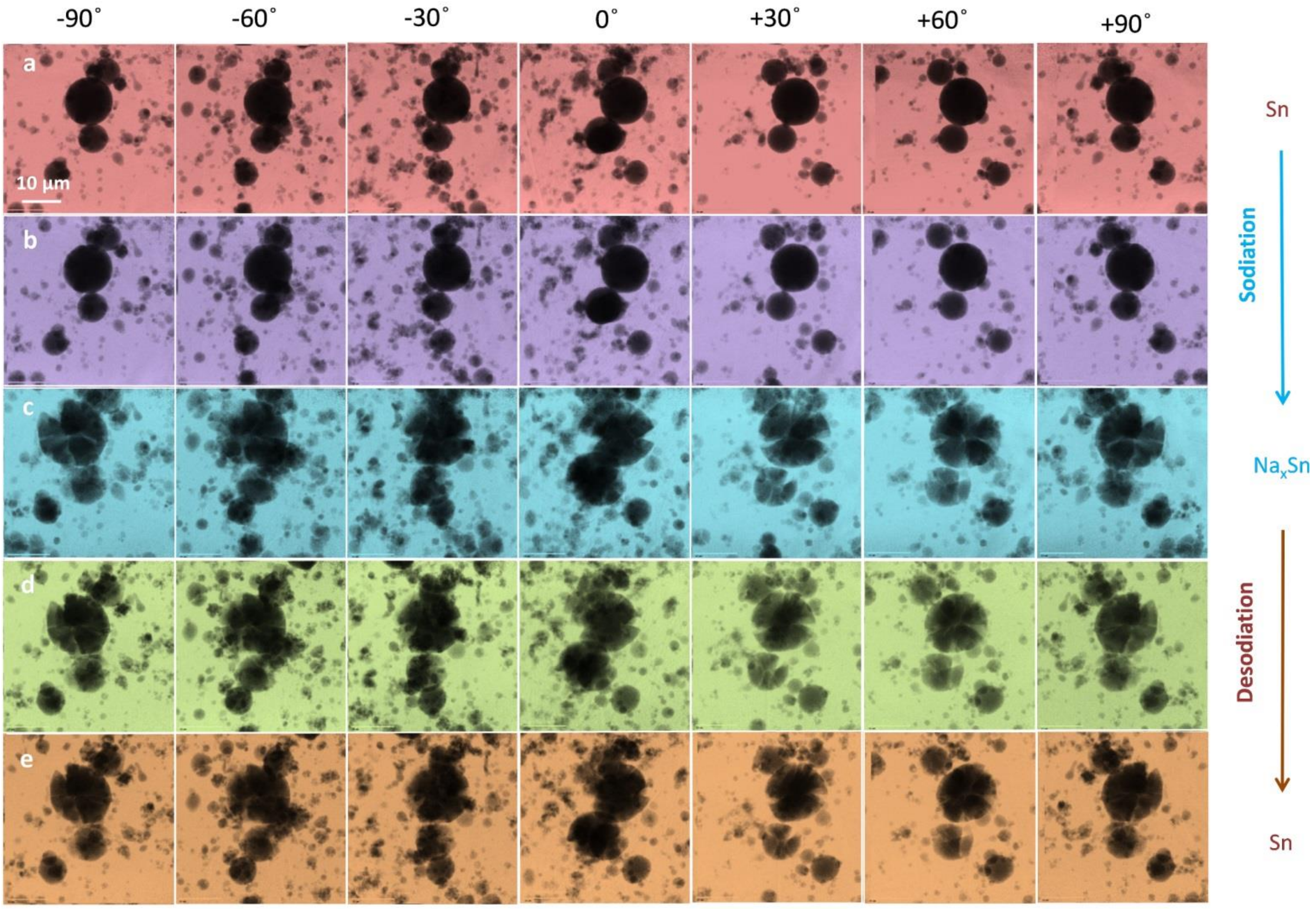

Supplementary Figure 1. 2D images of Sn anodes at different angles during the first sodiation-desodiation. (a) fresh; (**b**) partially sodiated; (**c**) sodiated; (**d**) partially desodiated; (**e**) desodiated. The electrochemical measurement was performed at a current density of 5 mA/g at the voltage range of 0.005-1.0V. After the initial sodiation in b, Sn particles show no change in morphology, but with further sodiation shown in C, cracks form and significant morphology change is observed. During the desodiation cycle (d-e), sodium ion extraction induces volume shrinkage, but the overall microstructure integrity with negligent pulverization. We demonstrate these results as evidence that mechanical degradation predominantly takes place at the first sodiation step (the alloying process).

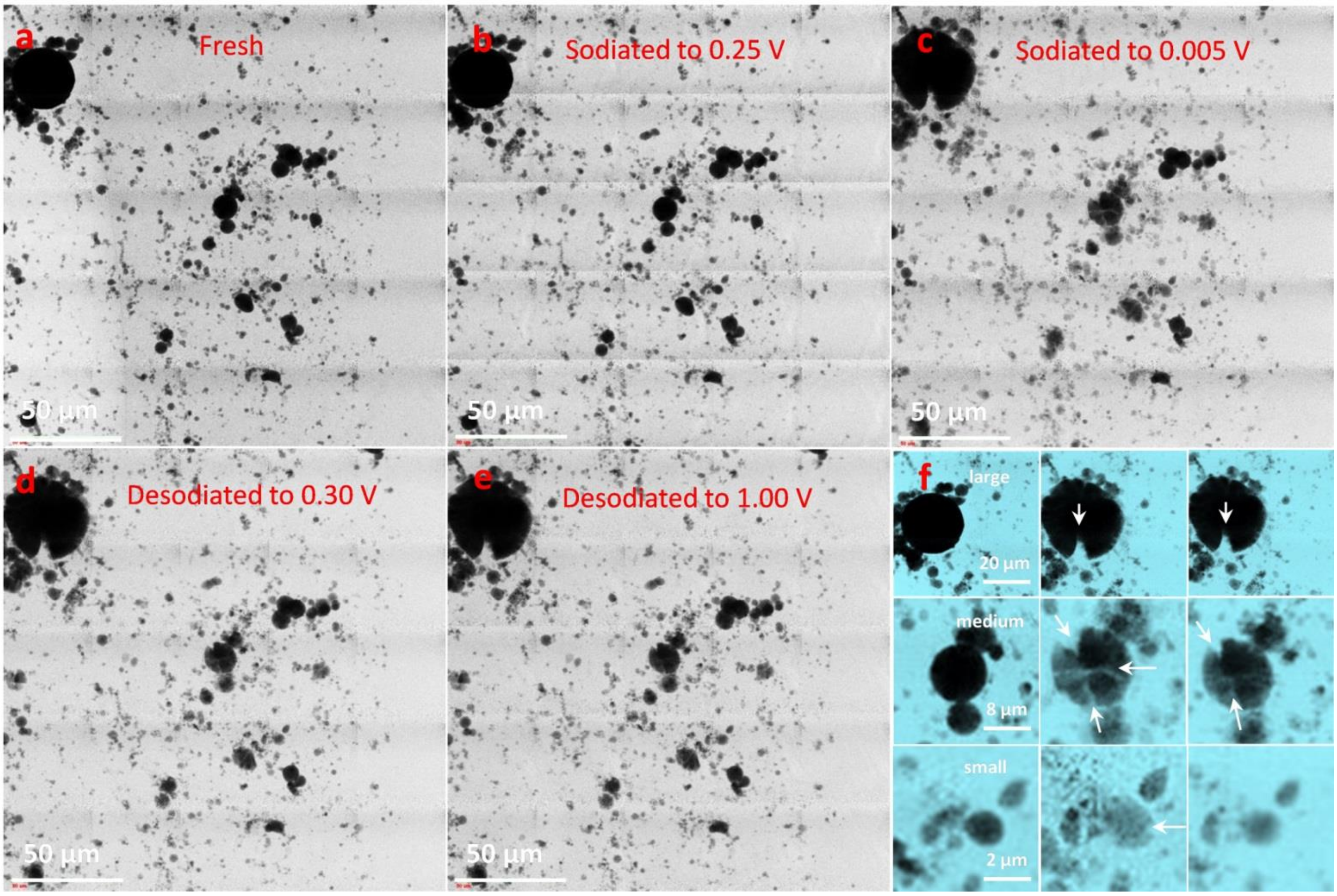

Supplementary Figure 2. 2D Mosaic images (5×5) of Sn anodes during the first sodiation-desodiation cycle in the mini Na-ion battery. (a) fresh; (**b**) partially sodiated; (**c**) sodiated; (**d**) partially desodiated; (**e**) desodiated. The electrochemical measurement was performed at a current density of 5 mA/g at a voltage range of 0.005-1.0V. Regardless of particle size, all of these Sn particles in this large-scale electrode (200×200 μm^2) show significant morphology change, indicating that the electrochemical sodiation/desodiation occurs throughout the entire electrode. (**f**) Morphological evolution of selected Sn particles with different sizes (large, medium and small) at the three electrochemical stages (fresh, sodiated to 0.005V and desodiated to 1.0 V). Most observed particles (big or small) show similar morphological features after the first sodaition and no obvious size dependent cracks was observed at this step, but a size-dependent mechanical reversibility seems occur at the following desodiation. As is observed when Na ions extraction, small particle shows significant volume shrinkage, whereas large particle exhibits negligent change. This phenomenon can be attributed to the low sodiation capacity and the lack of robust mechanical structure of large sized Sn particles.

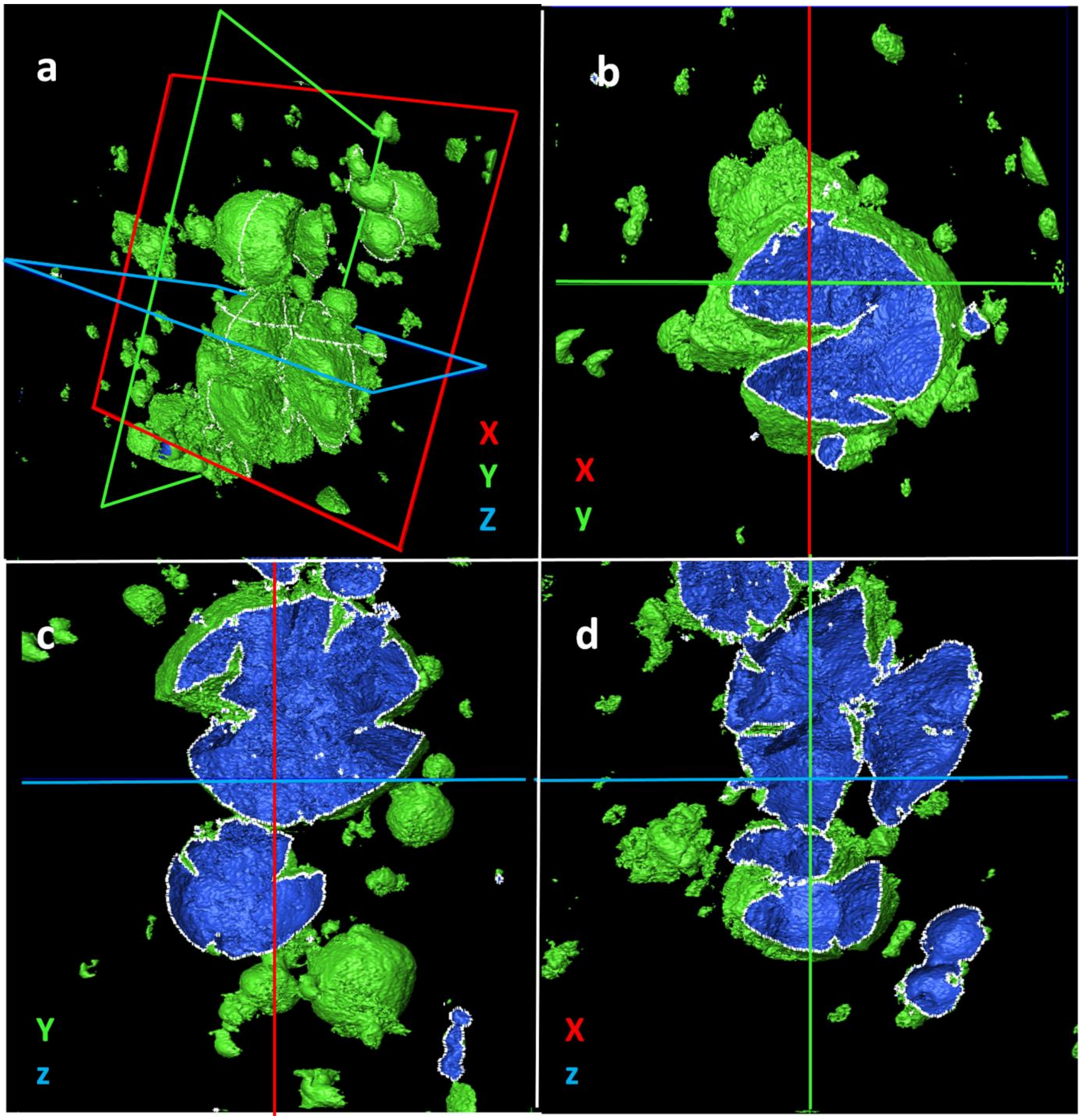

Supplementary Figure 3. 3D mechanical fracture of Sn anodes after the first sodiation viewed at different orientations . (a) the 3D overall morphology (x,y,z); (b) viewing along Z axis; (c) viewing from X axis; (d) viewing from Y axis. These different views unambiguously indicate the significant microstructural degradation after the first sodiation process.

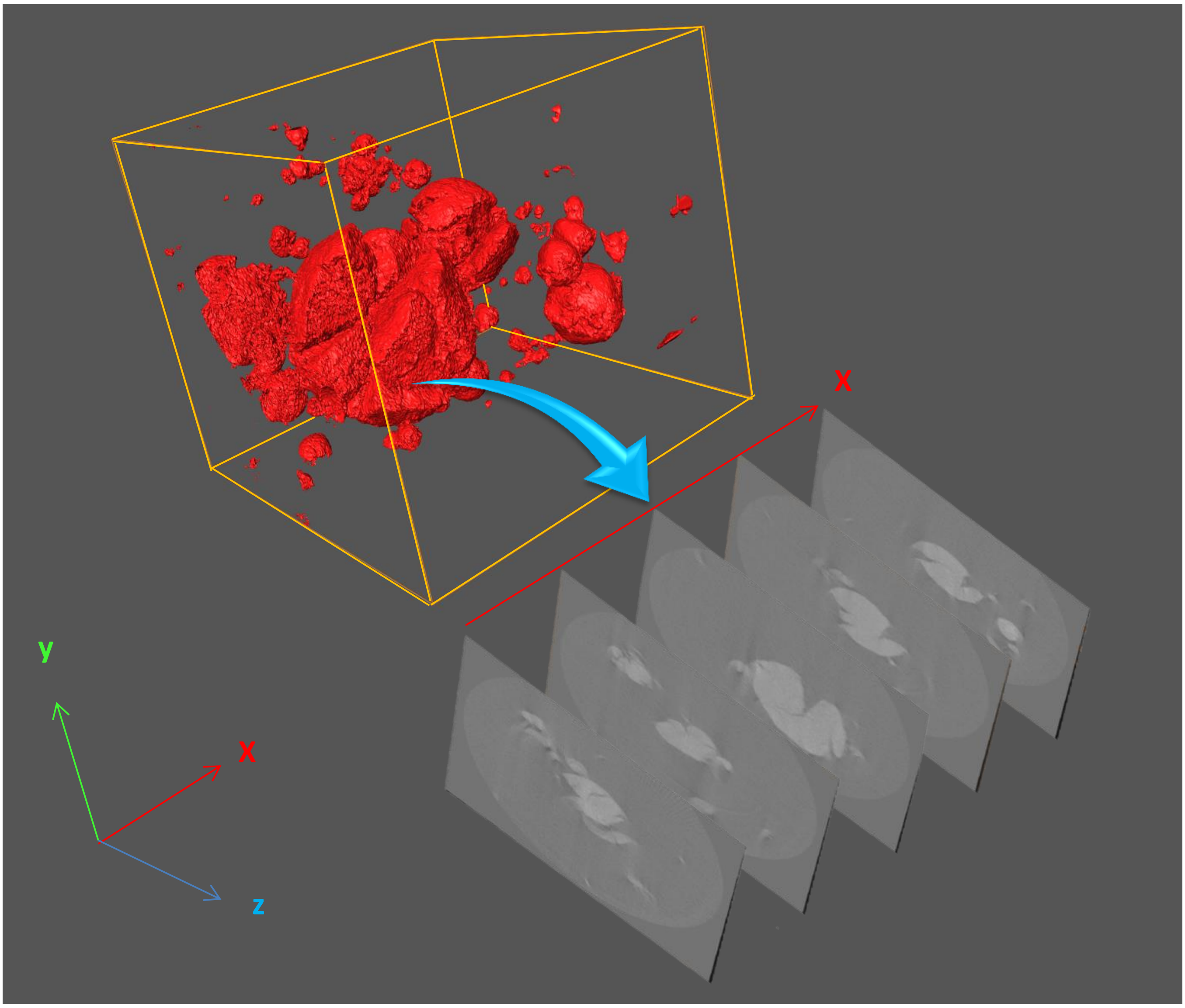

Supplementary Figure 4. The stacked cross-section images for the first sodiated Sn sample. The cross-section images were selected along X axis, showing the significant microstructural change from surface to inside the Sn particles.

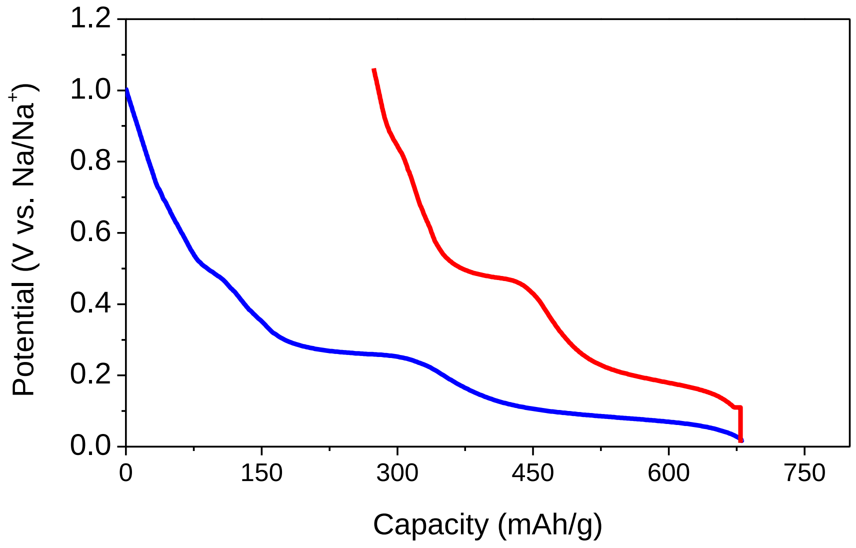

Supplementary Figure 5. The charge/discharge profile of Sn anodes in sodium-ion batteries duringthe first cycle. The electrochemical measurement was performed at a current density of 5 mA/g at the voltage range of 0.005-1.0V.

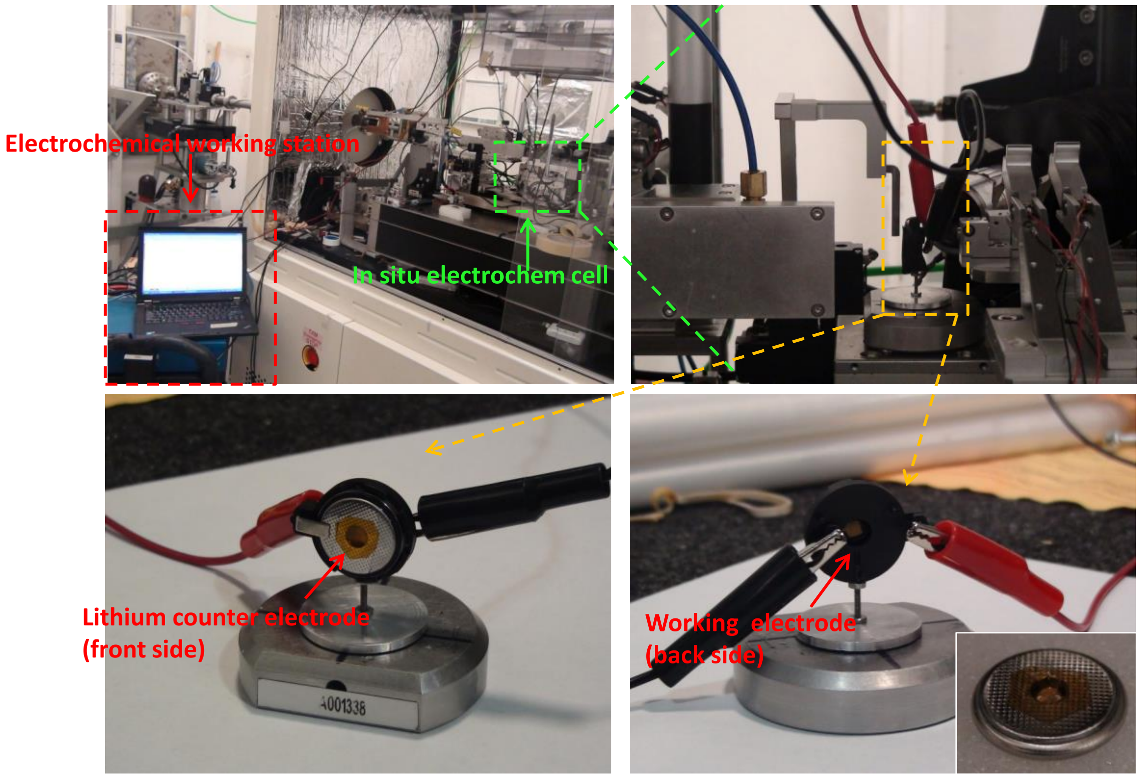

Supplementary Figure 6. The photo of the *in operando* 2D electrochemical cell setup at X8C, NSLS.

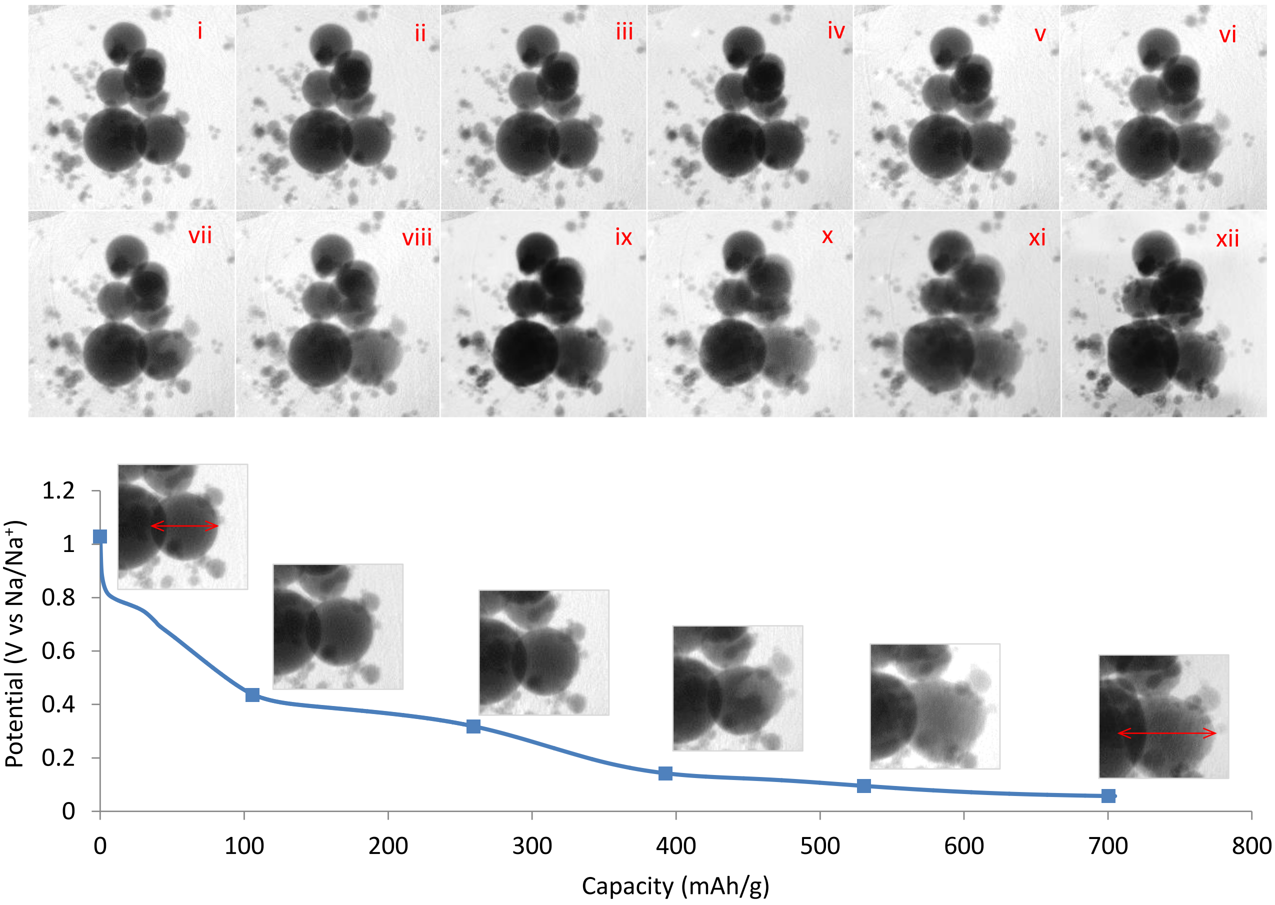

Supplementary Figure 7. The *in operando* 2D morphological evolution of Sn particles in Na-ion batteries. The electrochemical measurement was performed at a current density of 5 mA/g at the voltage range of 0.005-1.0 V.

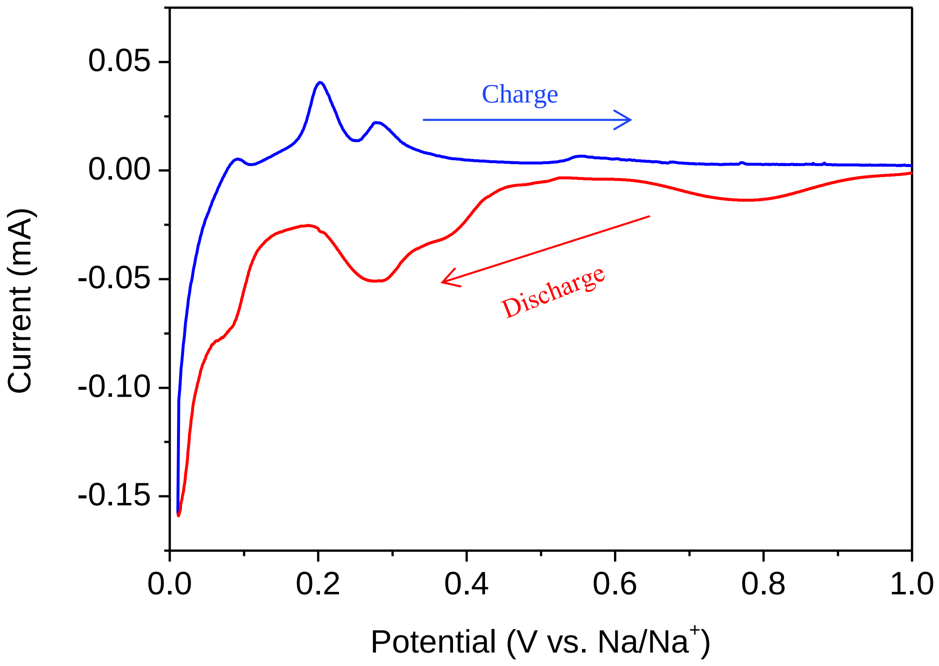

Supplementary Figure 8. The cyclic voltammetry measurement of Sn particles in Na-ion batteries. The electrochemical measurement was performed at the voltage range of 0.005-1.0 V.

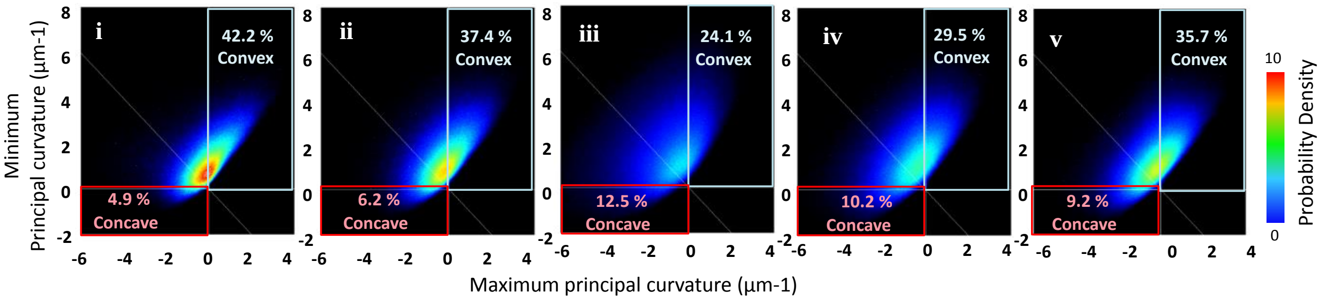

Supplementary Figure 9. Curvature distribution of Sn particles during the first sodiation-desodiation cycle.

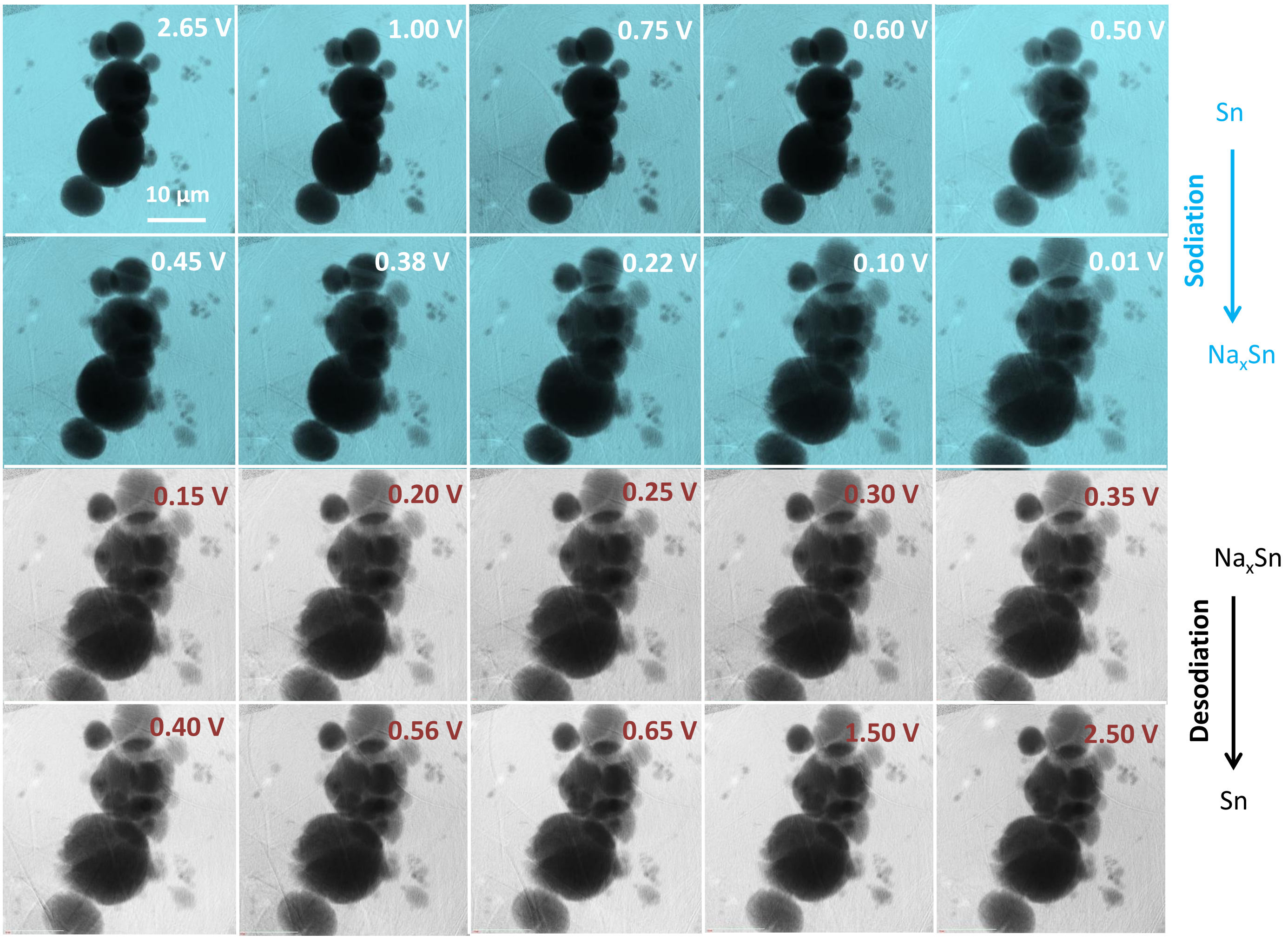

Supplementary Figure 10. The 2D morphological evolution of Sn electrode under *in operado* 2D TXM experiment. The electrochemical measurement was performed by cyclic voltammogram between 0.005 and 2.5 V at a scanning rate of 0.04 mV/s. Similar to the in situ 3D TXM experiment with galvanostatic cycle, large volume expansion and shrinkage occur after the first sodiation and desodiation process, respectively. In spite of this significant volume change, no obvious microstructural pulverization was found at the Na ion extraction process (desodiation). As a result, the first sodiation process plays a predominant role in Sn microstructural failure. This conclusion is consistent with the in situ 3D TXM result.

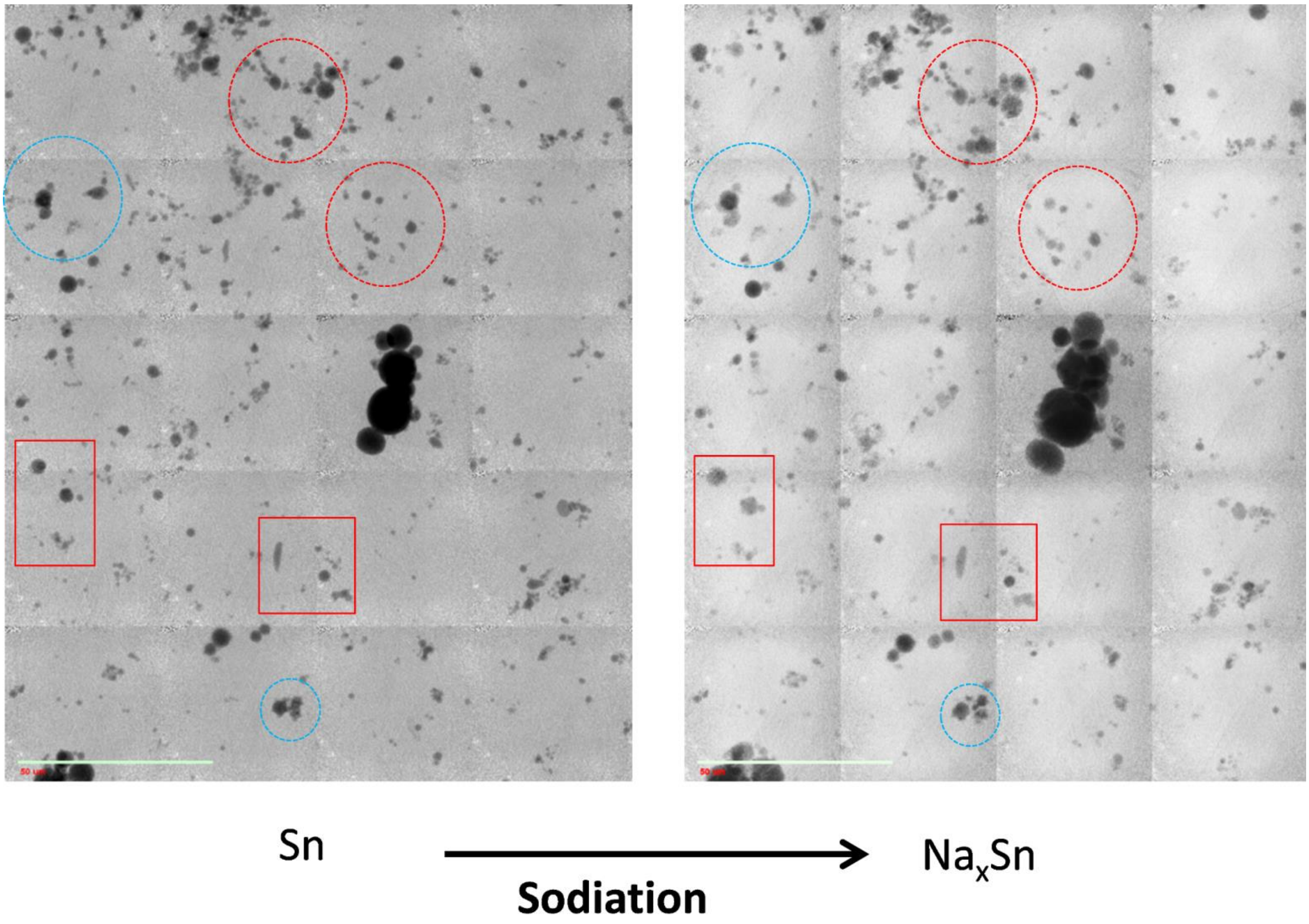

Supplementary Figure 11. The mosaic images (5×5) of the fresh (a) and 1st sodiated (b) Sn electrode under in operando TXM experiment. The electrochemical sodiation process occurs throughout the entire electrode, as significant volume expansion is shown in those marked regions. To clearly observe the morphology change, we select the larger particles at the central zone of the mosaic image which can reveal the representative microstructural evolution.

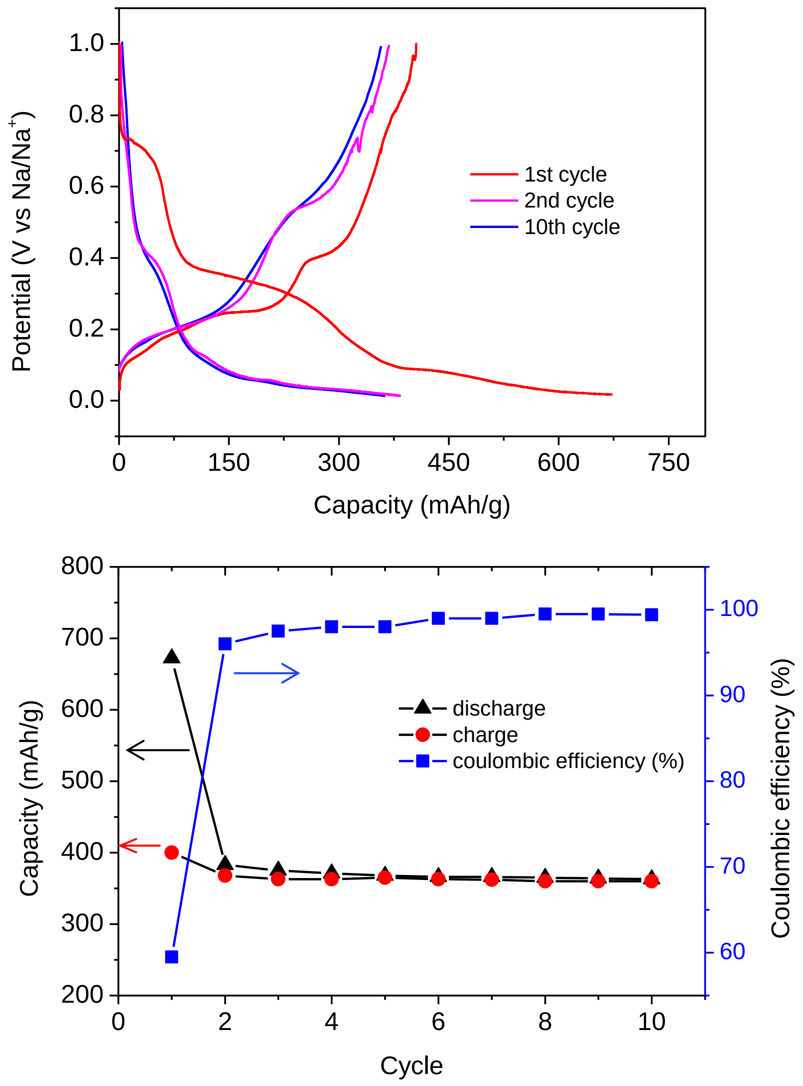

Supplementary Figure 12. Electrochemical performance of Sn anode in Na-ion batteries. (a) the discharge/charge profiles of Sn at the 1st, 2nd and 10th cycle at a current density of 5mA/g. (b) the specfic capacity and coulombic efficiency at the ten cycles.

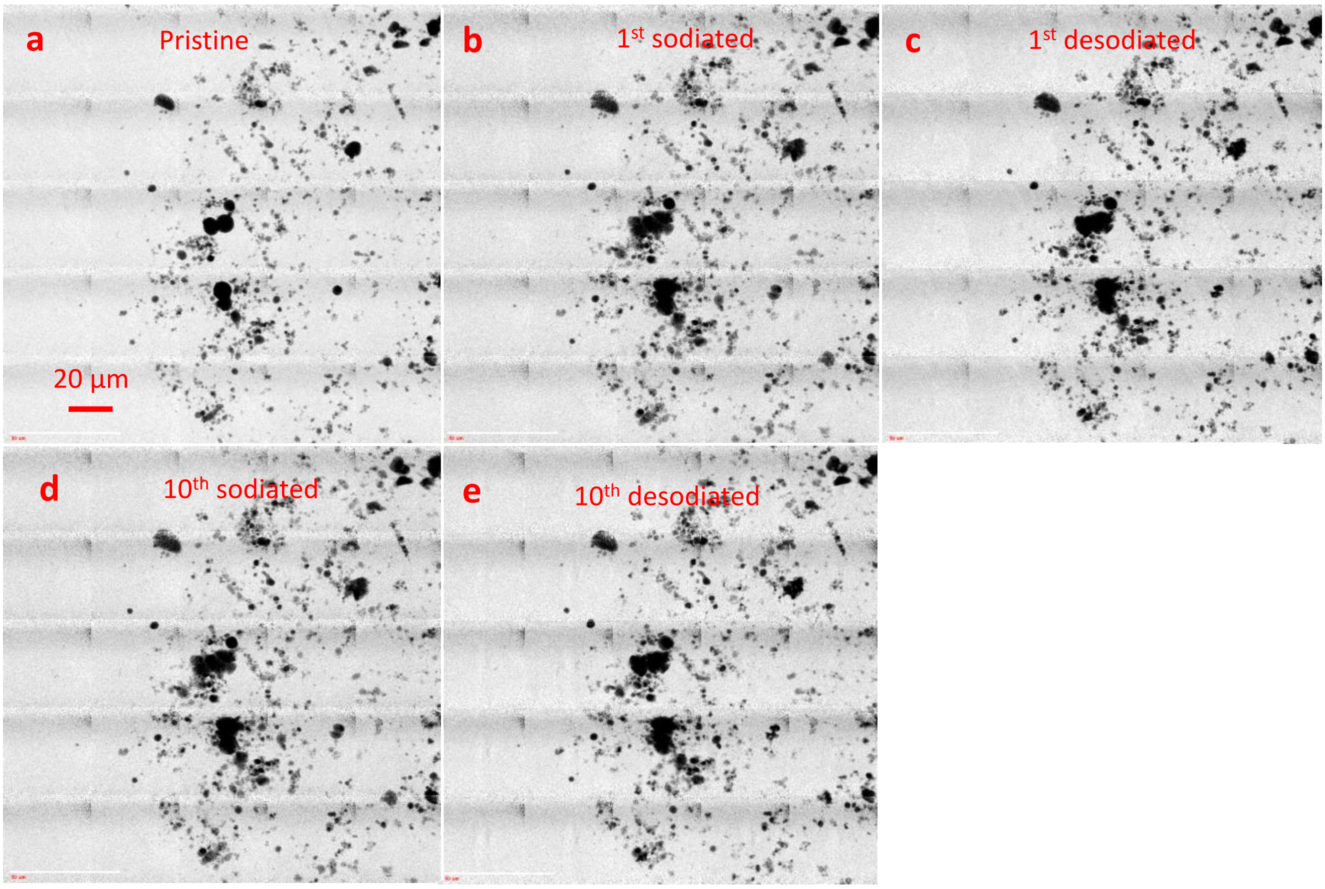

Supplementary Figure 13. 2D mosaic images of another Sn electrode during the 10 sodiation-desodiation cycle in the mini Na-ion battery. (a) Pristine; (**b**) 1st sodiated; (**c**) 1st desodiated; (**d**) 10th sodiated; (**e**) 10th desodiated. The electrochemical measurement was performed at a current density of 5 mA/g at a voltage range of 0.005-1.0V.

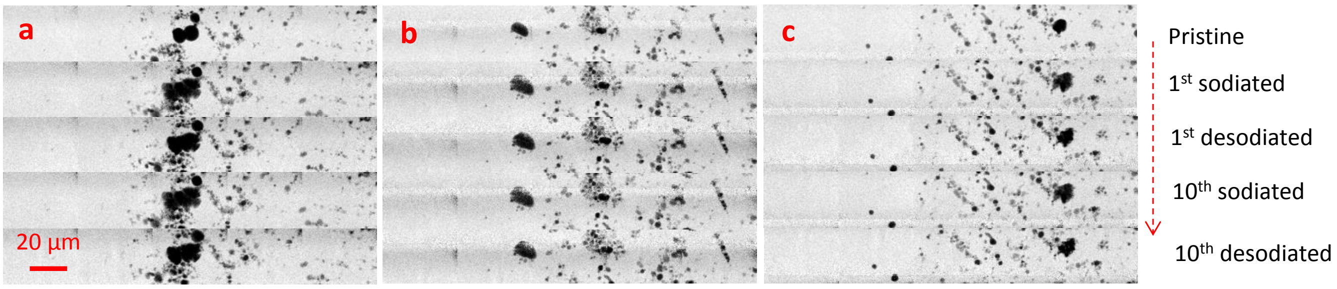

Supplementary Figure 14. Morphological evolution of selected zones at the Sn electrode during the 10 sodiation-desodiation cycle in the mini Na-ion battery. The electrochemical measurement was performed at a current density of 5 mA/g at a voltage range of 0.005-1.0V.

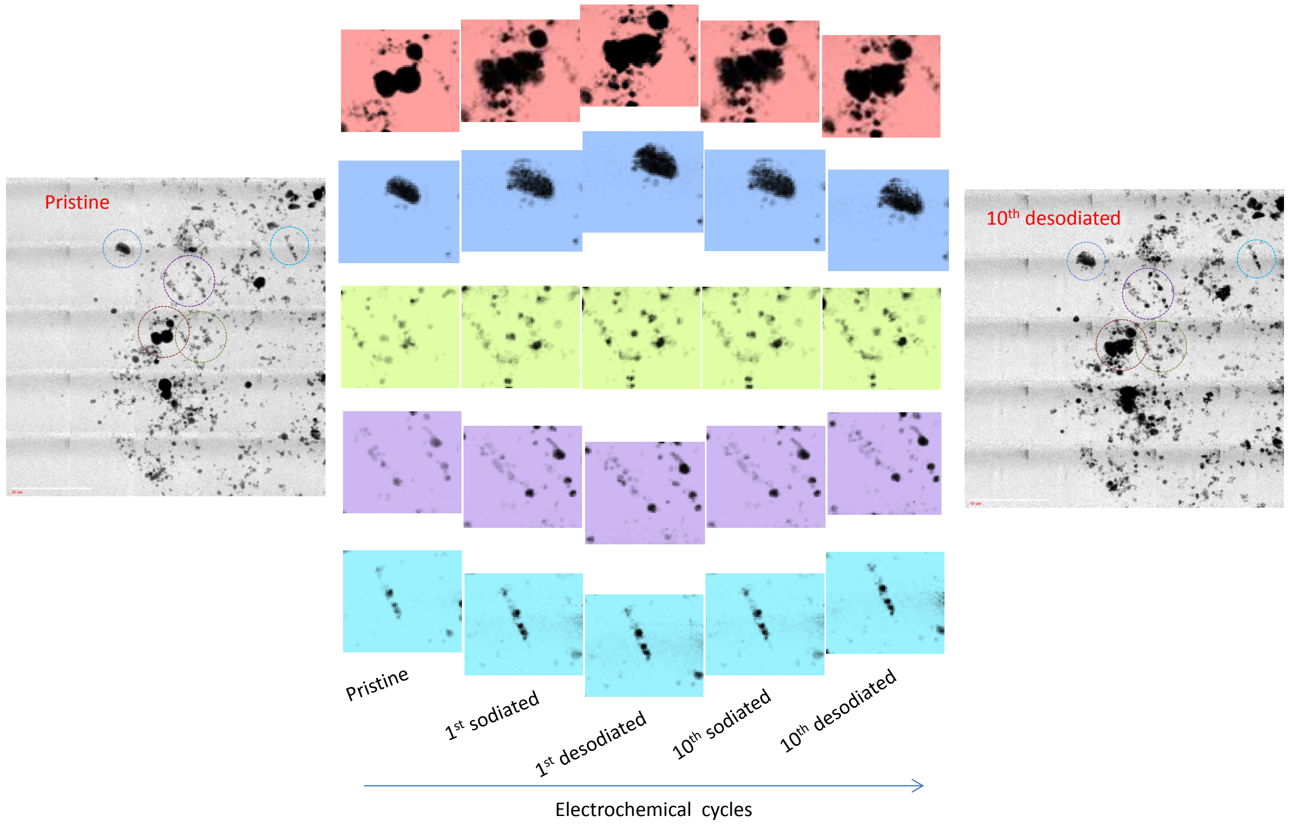

Supplementary Figure 15. Size effect obtained from the selected Sn particles with difference sizes during the 10 sodiation-desodiation cycle in the mini Na-ion battery. The electrochemical measurement was performed at a current density of 5 mA/g at a voltage range of 0.005-1.0V.

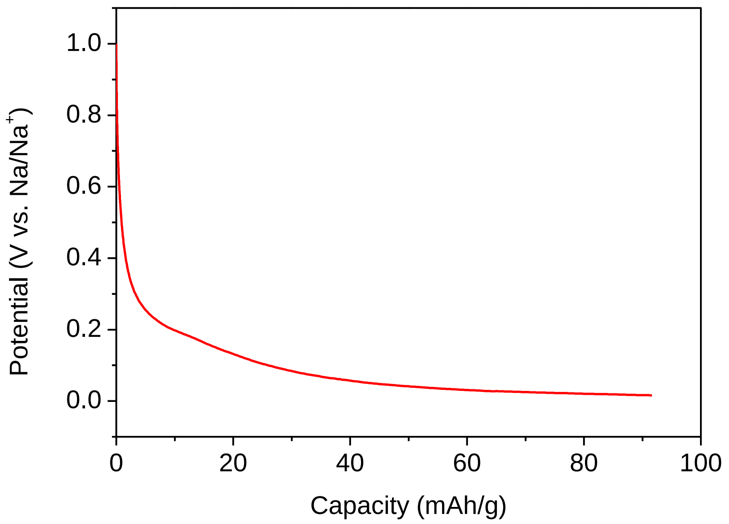

Supplementary Figure 16. The discharge profile of a pure carbon paper in Na-ion batteries. The electrochemical measurement was performed at a current density of 5 mA/g at the voltage range of 0.005-1.0 V.

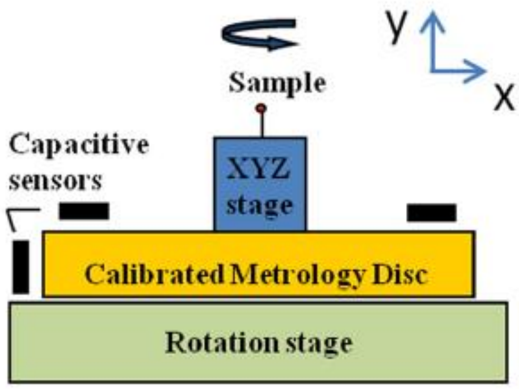

Supplementary Figure 17. A schematic showing the run-out correction system which consists of a precision metrology cylinder mounted on a low run-out ball bearing rotation stage and three integrated capacitive sensors to measure the residual run-out of the rotation axis as a function of rotation angle. ((Reprinted with permission from ref. 6. Copyright 2012 American Institute of Physics

Supplementary Tables

Electrochemical stages at the first cycle	i	ii	iii	iv	v
**Volume (nm^3, $\times10^{11}$)**	1.27	1.30	4.14	2.95	1.81
**Volume change (%) (compared to fresh)**	100	102	326	232	143
**Surface area (nm^2, $\times10^8$)**	2.24	2.26	11.94	8.56	5.27
**Surface area (%) (compared to fresh)**	100	101	533	382	235
**1/specific area (nm), S^{v-1}***	570.1	575.2	346.3	344.6	342.2
**Feature size change (%) (compared to fresh)**	100	100.1	60.7	60.4	60.0
**Concave feature (%)**	4.9	6.2	12.5	10.2	9.2
**Convex feature (%)**	42.2	37.4	24.1	29.5	35.7

Supplementary Table 1. Quantitative 3D morphological analysis of Sn anodes at the first cycle.

Electrochemical cycles	fresh	1st sodiation	1st desodiation	2nd sodiation	2nd desodiation	5th sodiation	5th desodiation	10th sodiation	10th desodiation
**Volume (nm^3, $\times10^{11}$)**	1.27	4.14	1.81	2.55	1.87	2.43	1.97	2.33	2.01
**Volume change (%) (compared to fresh)**	100	326	143	201	147	191	155	183	1.58
**Surface area (nm^2, $\times10^8$)**	2.24	11.94	5.27	7.59	5.62	7.50	6.07	7.22	6.21
**Surface area (%) (compared to fresh)**	100	533	235	338	251	335	271	322	277
**Specific area (μm^{-1}) S_v**	1.75	2.89	2.92	2.98	3.00	3.08	3.09	3.10	3.10
**Specific area change(%) (compared to fresh)**	100	165	167	170	171	176	177	177	177
**1/specific area (nm), S^{v-1}***	570.1	346.3	342.2	335.3	333.4	324.2	323.8	323.0	323.0
**Feature size change (%) (compared to fresh)**	100	60.7	60.0	58.8	58.5	56.9	56.8	56.7	56.7

Supplementary Table 2. Quantitative 3D morphological analysis of the electrodes at ten electrochemical cycles.

*specific area is defined as the surface area in per unit volume. While specific area is generally inversely proportional to the size, its reciprocal (1/specific area, S_v^{-1}) is then a suitable parameter used to indicate the representative size in a structure. Note that the precise relationship between the S_v^{-1} and the feature size in a system depends on sample's geometry. For instance, if the sample geometry can be represented by a sphere with a radius r, then the $S_v^{-1}$is:

$$\frac{\frac{4}{3}\pi r^3}{4\pi r^2} = \frac{r}{3}$$

Note that there is a geometric factor of 1/3, which is due to the spherical geometry.

Supplementary Notes

Supplementary Note 1. Challenges for in situ 3D electrochemical cells.

Developing such a working cell is very challenging because this cell must i) allow a 180-degree rotation without blocking x-ray beam; ii) fit micron scale studying electrode within the x-ray beam to fit within the x-ray field of view (40×40 μm); iii) function normally as a working battery; and iv) allow electrochemical measurement for correlating the microstructural changes with the electrochemical reaction stages. The widely used coin cell is insufficient because it blocks beam when it is rotated, leading to a very limited angle of rotation and producing an unusable 3D reconstruction. The supporting materials of the cell along the x-ray beam path must be highly transparent to allow sufficient transmission of the x-ray beam through the studied electrode. In addition, properly sealing such a cell is critical to ensure that the cell can work normally and be stable enough for repeated cycling. The complexity of developing the cell has hindered the investigation of in situ 3D microstructural evolution using TXM.

Supplementary Note 2. Electrochemical measurements.

Considering that Sn is difficult to maintain as fully sodiated to $Na_{15}Sn_4$ at high rates, particularly for the larger Sn particle size, we used an ultra-low current density of 5 mA/g to maximize the sodium ion insertion. The first galvanostatic cycle is between 0.005 to 1.0 V [1-3]. The initial high current pulse (done by pushing the cell voltage to be below 0.8 V) and the low cutoff voltage (1.0 V) strategies were applied to minimize the decomposition of electrolyte on Sn particles. In spite of this strategy, the electrolyte decomposition cannot be completely avoided. Therefore, to obtain the true capacity of Sn materials, a pure carbon paper (CP) electrode without Sn particles was tested with the same condition and the discharge profile was shown in Supplementary Figure 16. The discharge capacity is determined to be ~90 mAh/g (discharge to 0.005 V), which can be attributed to the carbon paper capacity and electrolyte decomposition. From the discharge-charge profile of Sn electrode, the overall discharge and charge capacity were determined to be 685 mAh/g and 405 mAh/g, respectively, with a coulombic efficiency of 59.1%, which is remarkably lower than most literature related to Sn in lithium-ion batteries [4,5], particularly considering the “minimization strategy” and the small capacity contribution (~90 mAh/g) from carbon paper and electrolyte decomposition. Therefore, this high irreversible capacity (~280 mAh/g) can be mainly attributed to the irreversibility in Na ions insertion/extraction due to the partial trapping of sodium ions in Sn electrodes, which can be further confirmed by the irreversibility at the above attenuation coefficient change and 3D microstructural evolution.

The discharge capacity of Sn at the second dataset point (~0.25 V) is determined to be 302 mAh/g, in which ~310 mAh/g (the entire Sn/CP electrode discharge capacity at ~0.25 V) substrates ~8 mAh/g (the CP electrode discharge capacity includes electrolyte decomposition at ~0.25 V). Therefore, the 302 mAh/g corresponds to a Na_xSn phase (x=~1.3).

The comparative study of Sn anodes in LIB was performed with the similar battery design and electrochemical methods. The only difference is using Li foil and 1M $LiPF_6$ in ethylene carbonate/diethyl carbonate (1:1) as counter electrode and electrolyte, respectively.

Supplementary Note 3. 3D morphological analysis method.

This system has a precision cylinder mounted on a low run-out ball bearing rotation stage and three integrated capacitive sensors as shown in schematic 1 to measure the residual run-out of the rotation axis as a function of rotation angle. These measured sensor readings are calibrated through multiple sets of tomography data from a standard gold ball (3 micron in diameter). Then, a program reads the calibrated measurements with an algorithm to obtain the run-out corrections $\Delta x(\theta)$ and $\Delta y(\theta)$ at each rotation angle as a function of the measured displacements of the capacitive sensors. When taking a tomography data for a sample, these run-out corrections are automatically applied to produce a transparent mode of usage that emulates a perfect system without any rotational run-out. Without any need for user interaction, each collected 2D projection is automatically aligned for 3D reconstruction to fulfill automated 3D tomography (Supplementary Figure 17).[6]

A standard Filtered Back-projection Reconstruction algorithm was again used to reconstruct the 3D images. The reconstructed volumes were cylinders with 40 μm in both diameter and height. The volumes from different electrochemical cycle states were then registered using commercial software (Avizo, VSG, version 7). A median filter with a kernel size of 3×3×3 voxels was then applied to the original image for noise reduction. The Sn/Na_xSn and exterior regions (electrolyte and carbon fiber) were labeled via simple threshold segmentation. The histogram of the reconstruction images consist of two distinctive peaks for these two phases and therefore the threshold value can be chosen as the minimum value between the two peaks. A smoothed surface mesh of the Sn/Na_xSn particles was then generated from the segmented images also using Avizo with a constraint that preserves the particle volumes within the surface meshes.

Various 3D parameters were then calculated from the segmented structure and surface meshes: particle feature size distribution, volume change, surface area, specific area, and curvature analysis. The volume change was calculated by voxel counting. The surface area was measured from the surface mesh. Specific area is defined as surface area per unit volume. It was calculated from dividing the surface area of all particles in the entire sample by the volume of all particles in the entire sample. It is a direct indication of the size change of the sample. A smaller particle of the same shape has larger specific area than a larger particle with the same shape. Therefore, the decrease of specific area indicates the morphological change

such as fracture, cracking and pulverization which all lead to increase the surface area while the total volume remains constant. The reciprocal of the specific area was then a common parameter used to characterize the average feature size.

The feature size distribution was calculated using software deFveloped in-house (MatLab, R2011b, MathWorks) with the algorithms described elsewhere by Holzer et al. The principal curvature calculations were carried out using commercial package (Avizo, v.7, VSG) [7]. The interfacial shape distribution (ISD) was then plotted using customized written software (MatLab, R2011b, MathWorks) with method developed by Voorhees et al.[8] In the ISD calculation, as the surface meshing in Avizo results in a triangular mesh with tiles of various areas, an area weighting procedure is used when generating the probability map.

Surface curvedness computes the surface scalar field which value are equal to

$$\frac{1}{2}\sqrt{C1^2 + C2^2} \quad (1)$$

Where C1 and C2 are the two principal curvatures.

Supplementary Note 4. Analysis of fracture degree.

The fracture degree (a unit-less parameter, η) is measured by the ratio of R_s to R_v. Here the R_s is the radius calculated from the actual surface area S,

$$S=4\pi R_s^2 \quad (2)$$

$$R_s = \sqrt{\frac{S}{4\pi}} \quad (3)$$

and the R_v is the radius from the actual volume V

$$V = \frac{4}{3}\pi R_v^3 \quad (4)$$

$$R_v = \sqrt[3]{\frac{3V}{4\pi}} \quad (5)$$

So, the fracture degree, η, can be determined below,

$$\eta=\frac{R_s}{R_v}=\frac{\sqrt{\frac{S}{4\pi}}}{\sqrt[3]{\frac{3V}{4\pi}}} \quad (6)$$

Here, the S and V are the 3D statistical analysis results based on hundreds of particles in the electrode.

For example in the ideal case of the ball-shaped particle, if a particle has no fracture during sodiaton-desodiation, the above R_s is equal to R_v, so the fracture degree for the particle is around 1.

Supplementary References

1. Ellis, L. D., Hatchard, T. D., Obrovac, M. N. Reversible insertion of sodium in tin. *J. Electrochem. Soc.* **159**, A1801-A1805 (2012).
2. Hatchard, S. D. T., Bonakdarpour, A., Hewitt, K. C., Dahn, J. R. Anomalous, high-voltage irreversible capacity in tin electrodes for lithium batteries. *J. Electrochem. Soc*. **150**, A701-A705 (2003).
3. Courtney, I. A., Dahn, J. R. Electrochemical and in situ X-ray diffraction studies of the reaction of lithium with tin oxide composites. *J. Electrochem. Soc*. **144**, 2045-2052 (1997).
4. Idota, Y., Kubota, T., Matsufuji, A., Maekawa, Y., Miyasaka, T. Tin-based amorphous oxide: a high-capacity lithium-ion-storage material. *Science* **276**, 1395-1397 (1997).
5. Luo, B. *et al*. Graphene-confined Sn nanosheets with enhanced lithium storage capability. *Adv. Mater*. **24**, 3538-3543 (2012).
6. Wang, J. et al. Automated markerless full field hard x-ray microscopic tomography at sub-50?nm 3-dimension spatial resolution. *Appl. Phys. Lett*. **100**, 143107 (2012).
7. Munch, B., Holzer, L. Contradicting geometrical concepts in pore size analysis attained with electron microscopy and mercury intrusion. *J. Am. Ceram. Soc.* **91**, 4059-4067 (2008).
8. Alkemper, J., Voorhees, P. W. Three-dimensional characterization of dendritic microstructures. *Acta Mater*. **49**, 897-902 (2001).
